# Edges of Saturn’s rings are fractal

**DOI:** 10.1186/s40064-015-0926-6

**Published:** 2015-04-01

**Authors:** Jun Li, Martin Ostoja-Starzewski

**Affiliations:** Division of Engineering and Applied Science, California Institute of Technology, 91125 Pasadena, CA USA; Department of Mechanical Science & Engineering, Institute for Condensed Matter Theory and Beckman Institute, University of Illinois at Urbana-Champaign, 61801 Urbana, IL USA

**Keywords:** Saturn’s rings, Fractals, Cassini mission, Ring divisions

## Abstract

The images recently sent by the Cassini spacecraft mission (on the NASA website http://saturn.jpl.nasa.gov/photos/halloffame/) show the complex and beautiful rings of Saturn. Over the past few decades, various conjectures were advanced that Saturn’s rings are Cantor-like sets, although no convincing fractal analysis of actual images has ever appeared. Here we focus on four images sent by the Cassini spacecraft mission (slide #42 “Mapping Clumps in Saturn’s Rings”, slide #54 “Scattered Sunshine”, slide #66 taken two weeks before the planet’s Augus’t 200’9 equinox, and slide #68 showing edge waves raised by Daphnis on the Keeler Gap) and one image from the Voyager 2’ mission in 1981. Using three box-counting methods, we determine the fractal dimension of edges of rings seen here to be consistently about 1.63 ~ 1.78. This clarifies in what sense Saturn’s rings are fractal.

## Background

**Figure 1:**
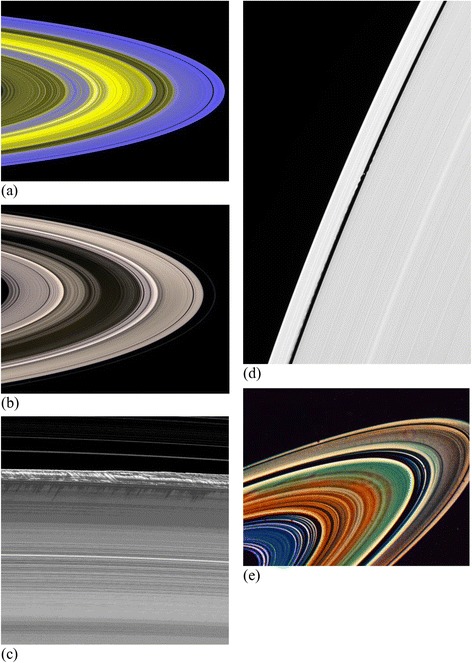
**(a,b,c,d,e): The original images of the Cassini and Voyager missions.**

The images recently sent by the Cassini spacecraft mission (available on the NASA website http://saturn.jpl.nasa.gov/photos/halloffame/) show the complex and beautiful rings of Saturn. Beginning with (Mandelbrot, 1982; Avron and Simon, 1981; Fridman and Gorkavyi, 1994), there have been conjectures that radial cross-sections of Saturn’s rings are Cantor sets, but, to the best of our knowledge, no convincing fractal analyses of actual images ever appeared. Of the 87 Cassini images, in Figure 1 (a) we reproduce slide #42 bearing the title “Mapping Clumps in Saturn’s Rings,” in Figure 1 (b) the slide #54 titled “Scattered Sunshine,” in Figure 1 (c) we reproduce slide #66 taken two weeks before the planet’s August 2009 equinox, and in Figure 1 (d) slide #68 showing edge waves raised by Daphnis on the Keeler Gap. The first of these is a false-color image of Saturn’s main rings made by combining data from multiple star occultations using the Cassini ultraviolet imaging spectrograph. In the second of these, Saturn’s icy rings shine in scattered sunlight, from about 15° above the ring plane. In the third image, a part of the Cassini Division, between the B and the A rings, appears at the top of the image, showing ringlets in the inner division, while in the fourth Daphnis cruises through the Keeler Gap, raising edge waves in the ring material as it passes. The first two photographs show the curved geometry of Saturn’s main rings with a low opening angle, while the latter two reflect the details of a part of the rings. Finally, in Figure 1 (e), we reproduce the image sent by ‘Voyager 2’ spacecraft in 1981 (http://solarsystem.nasa.gov/planets/images/inset-saturn-rings-large.jpg). The selected set of images represent Saturn’s rings from a variety of view angles and regions.

## Results and discussion

**Figure 2:**
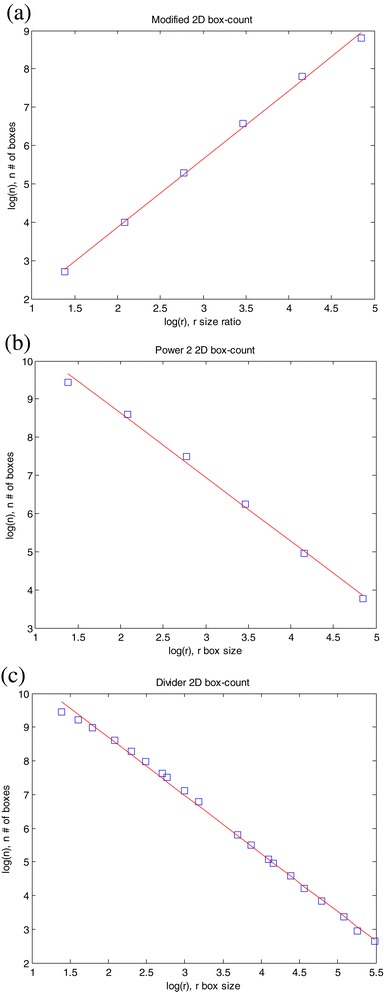
**Results of box counting method to estimate the fractal dimension of image (a) in Figure** **1** **: (a) Modified box counting; (b) Power 2 box counting; (c) Divider box counting.**

**Figure 3:**
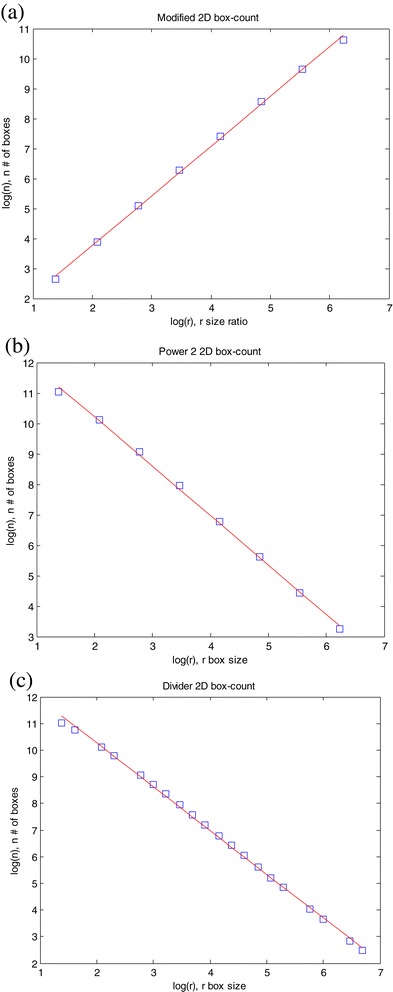
**Results of box counting method to estimate the fractal dimension of image (d) in Figure** **1** **: (a) Modified box counting; (b) Power 2 box counting; (c) Divider box counting.**

**Figure 4:**
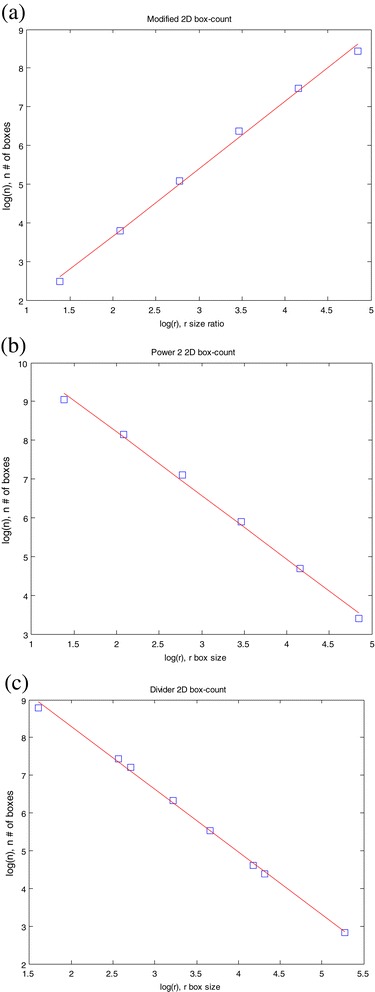
**Box counting method to estimate the fractal dimension of image (e) in Figure** **1** **: (a) Modified box counting; (b) Power 2 box counting; (c) Divider box counting.**

As is well known (Mandelbrot, 1982), the fractal dimension *D* comes from estimation of the slope of log(*n*)-log(*r*) in *n* ∝ *r*^− *D*^, where *n* is the number of boxes with size *r* needed to cover the region of interest. The local slopes of log(*n*)-log(*r*) are also acquired to determine optimal cut-offs of box sizes. The cut-offs are specified where the local slope varies strongly. The log(*n*)-log(*r*) plots of the three box counting methods for images of Figure 1 (a), (d), and (e) are shown in Figures 2, 3 and 4, respectively. Since the plots for Figures 1 (b) and (c) are very similar to the others, they are not shown here in order to save space. Note that, for modified box counting, *r* denotes the ratio of image size to box size, unlike power 2 or divider box counting, where *r* is the box size.

Note that these images were projections of Saturn’s rings from different angles and regions. Following the arguments presented in (Maggi 2008; Meakin 1998), given the fact that the rings’ thickness is extremely small compared to their radii, the projection onto the plane of the photograph does not affect the fractal dimension. Besides, the self-similarity of fractals indicates that the fractal dimension of a part is same as that of the whole. Overall, the box counting results of all images are given in Table 1.

**Table 1:** **Box counting results**

**Image sources**	**Modified box counting**	**Power 2 box counting**	**Divider box counting**
Figure 1. (a)	1.63	1.65	1.66
Figure 1. (b)	1.64	1.65	1.71
Figure 1. (c)	1.78	1.71	1.76
Figure 1. (d)	1.64	1.74	1.66
Figure 1. (e)	1.67	1.72	1.77

## Conclusions

All the images analyzed in this paper yield fractal dimensions in the range 1.63 to 1.78. This is a consistent estimate of the fractal dimension of the rings’ edges, regardless of the various image sources we utilized. Indeed, the fact that the rings’ edges are fractal provides one more hint to developing models of the intricate mechanics and physics governing these structures of granular matter. Interestingly, somewhat related studies (Feitzinger and Galinski 1987; de la Fuente and de la Fuente 2006a, b) found average fractal dimension ~1.7 for the projected fractal dimension of the distribution of star-forming sites (HII regions) in a sample of 19 spiral galaxies.

## Methods

**Figure 5:**
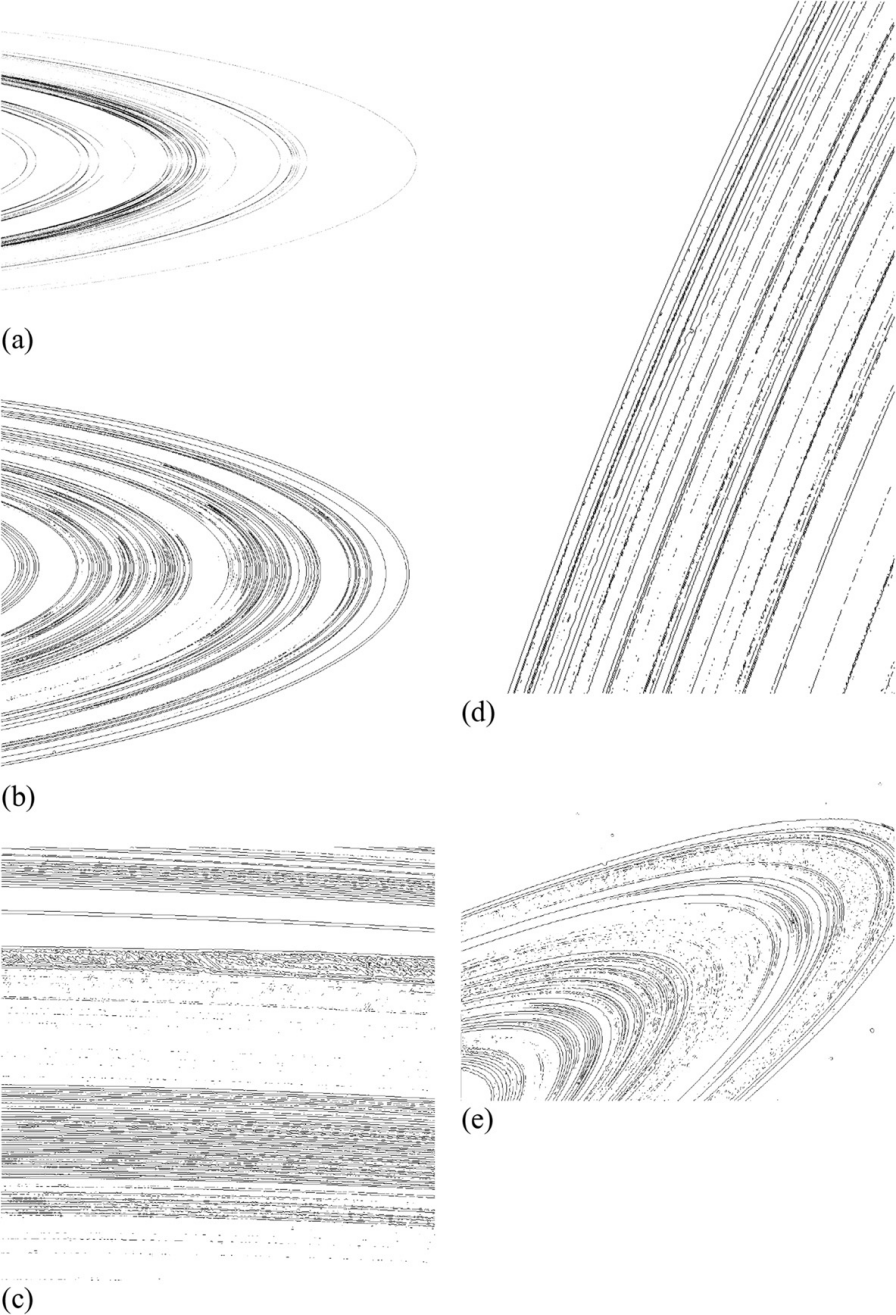
**(a,b,c,d,e): Images processed, respectively, from Figure** **1** **(a,b,c,d,e) to capture the ring edges.**

Using the box counting method, we determine the fractal dimension of edges of those rings. First, various edge detection methods are performed and compared to optimally identify ring boundaries: ‘Sobel’, ‘Robert’, ‘Laplacian of Gaussian’, ‘Canny’ and ‘Zero-Cross’ edge functions in the Matlab Image Processing Toolbox. Furthermore, the morphology operation functions of ‘bridge’, ‘close’, ‘thicken’, ‘thin’ and ‘skel’ are employed to connect some isolated pixels and also remove redundant pixels on the boundaries from consideration of physical reality. It was found that the option of ‘Laplacian of Gaussian’ edge function with ‘close’ and ‘thin’ morphology operation produced optimal appearance of ring boundaries. The resulting edge images are displayed in Figure 5 (a-e), respectively, for the five original images we displayed in Figure 1 (a-e).

We perform three box counting methods to estimate fractal dimensions of the above processed black-white images of Saturn rings, so as to take into account the influences of the sizes and shapes of covering boxes:

1. Modified box counting using boxes with shape being self-similar to the global image. This method is well suited for generally rectangular images (Xu and Lacidogna 2011), where the boxes are rectangles self-similar to the whole image. The selection of the ratio of image size to box size is in powers of 2 for optimal log(*n*)-log(*r*) regression. When the ratio does not give an integer box size, the box size was chosen to be the closest integer at that ratio.
2. Power 2 box counting using boxes with sizes as powers of 2, possessing optimal log(*n*)-log(*r*) regression. Here the partial boarder effects are evident generally when the image size was not powers of 2. In this case the image was embedded in an empty image with size being powers of 2 closest to the original image size. The box counting was then performed on the ‘enlarged’ image.
3. Divider box counting using boxes with sizes being the dividers of the image size. Subsequent box size may be too close for log(*n*)-log(*r*) regression, while the border effects can be eliminated.

**Figure 6:**
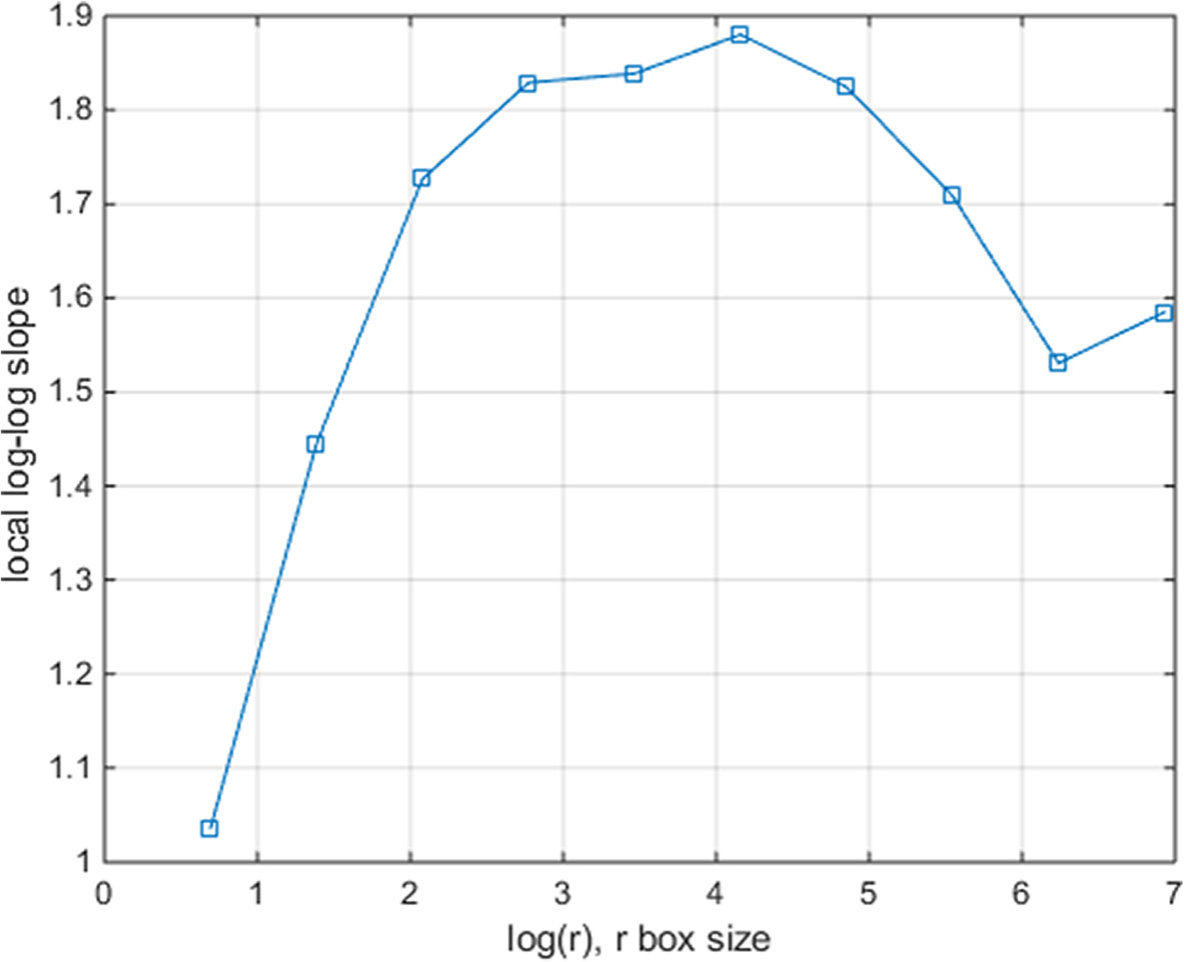
**An example of the local slope of log(n)-log(r) for power 2 box counting applied to Figure** **1** **(a) with r=2 to r=b/2, where b denotes the image size (extended to powers of 2).**

In particular the cut-offs of box sizes are considered by examining the local slopes of log(*n*)-log(*r*). Figure 6 shows an example of the local slope of log(*n*)-log(*r*) for power 2 box counting applied to Figure 1 (a) with *r* = 2 to *r* = *b*/2, where *b* denotes the image size (after extended to powers of 2). The fine box size *r* = 2 tends to be below the average spacing of ring particles, whereas the very coarse box count (*r* = *b*/2) usually fails to capture structural details. The lower and upper cut-offs of box sizes are then 4 and b/4.
